# Gamma Oscillations Facilitate Effective Learning in Excitatory-Inhibitory Balanced Neural Circuits

**DOI:** 10.1155/2021/6668175

**Published:** 2021-01-20

**Authors:** Kwan Tung Li, Junhao Liang, Changsong Zhou

**Affiliations:** Department of Physics, Centre for Nonlinear Studies and Beijing-Hong Kong-Singapore Joint Centre for Nonlinear and Complex Systems (Hong Kong), Institute of Computational and Theoretical Studies, Hong Kong Baptist University, Kowloon Tong, Hong Kong

## Abstract

Gamma oscillation in neural circuits is believed to associate with effective learning in the brain, while the underlying mechanism is unclear. This paper aims to study how spike-timing-dependent plasticity (STDP), a typical mechanism of learning, with its interaction with gamma oscillation in neural circuits, shapes the network dynamics properties and the network structure formation. We study an excitatory-inhibitory (E-I) integrate-and-fire neuronal network with triplet STDP, heterosynaptic plasticity, and a transmitter-induced plasticity. Our results show that the performance of plasticity is diverse in different synchronization levels. We find that gamma oscillation is beneficial to synaptic potentiation among stimulated neurons by forming a special network structure where the sum of excitatory input synaptic strength is correlated with the sum of inhibitory input synaptic strength. The circuit can maintain E-I balanced input on average, whereas the balance is temporal broken during the learning-induced oscillations. Our study reveals a potential mechanism about the benefits of gamma oscillation on learning in biological neural circuits.

## 1. Introduction

The emergence of oscillations in neurophysiological signals within different frequency bands is commonly observed in mammal brain [1–4]. This cross-species phenomenon is behaviorally relevant. When animals or humans are performing different tasks or at the resting state, neural oscillations within specific frequency bands would be detected in specific brain regions. For example, alpha oscillations can be detected in occipital electroencephalography (EEG) signal when humans are in an eye-closed state, but it transitions to beta band when eyes are open [1].

Learning is the ability of acquiring new knowledge, behaviors, skills, attitudes, preferences, etc. possessed by humans and animals [5]. Physiologically, learning is believed to accompany with structural changes of neural circuits in the brain and is frequently observed to associate with the emergence of gamma band oscillations [6–8]. Through learning processes, structural clusters are formed in neural networks in both the prefrontal cortex and hippocampus, which are behaviorally relevant for the recall of short-term memory and the later consolidation of long-term memory [9, 10].

Although it is commonly accepted that gamma oscillations emerge during learning, their relation is not yet clear. First, the mechanism underlying gamma oscillation in neural circuits is still under active debate [8, 11–15]. Second, the learning process in the neural circuit level is often phenomenally explained through plasticity mechanism [16–18], while plasticity mechanisms in neural circuits are diverse and their effects are elusive. Especially, how plasticity interacts with neural dynamics is not well understood.

In this paper, we study learning in neural networks through spike-timing-dependent plasticity (STDP), a widely-observed phenomenon in experiments. It describes how synaptic strength changes according to the spiking time difference of neurons. STDP has become one of the learning rules widely used for studying cluster formation in simulating learning process in neural networks [19, 20]. Triplet STDP rule, heterosynaptic plasticity, and other plasticity rules are used together in Zenke et al. [19] to study cell assemblies and memory recall. Zenke et al. [19] and previous studies showed that triplet STDP rule can account for firing frequency dependence of spiking [19, 21], which is not considered in traditional pair-based STDP rule [22, 23], and heterosynaptic plasticity can prevent explosive increase of synaptic weight [24, 25]. Excitation-inhibition neural circuit with synaptic kinetics can allow fast network oscillation with a wide range of frequency [6, 7, 11, 26–28]. Under suitable network parameters, such networks can generate gamma oscillations. In recent studies, gamma oscillation has been shown beneficial for coding input signal through plasticity [29, 30], inducing the postneurons to phase lock with the input signal [31], and is stable for transferring multiplexing signal [32]. However, these studies focus on plasticity between external inputs and receiving neurons. How gamma oscillation interacts with plasticity to form a stable cluster within a recurrent circuit is not well understood. Here, we used excitation-inhibition circuit of integrate-and-fire neurons with synaptic kinetics to study the effects of plasticity including STDP, heterosynaptic plasticity, and transmitter-induced plasticity. We aimed to study how gamma oscillation is generated and how it interplays with plasticity during the learning process.

**Figure 1:**
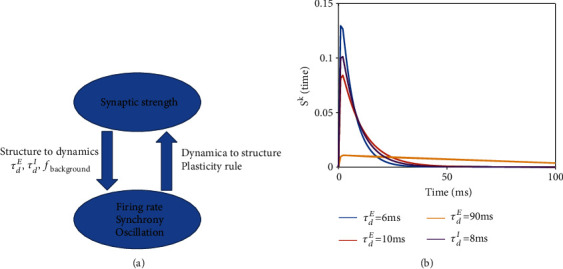
Schematic diagram of the study. (a) A paradigm of the interaction between network structure and dynamics. Branch I (left): synaptic weights influence the circuit dynamical properties, and this influence depends on other dynamical parameters such as synaptic time constant (*τ*_*d*_^*E*^, *τ*_*d*_^*I*^) and background input *f*_background_. Branch II (right): the circuit dynamics influence the synaptic weights through plasticity rule. (b) Illustration of the synaptic time course *S*^*k*^(*t*), *k* = *E*, *I* in Eq.(3) with different decay time *τ*_*d*_^*k*^.

In neural networks with plasticity, the neural circuit dynamic properties and the network structure (synaptic strength) interact with each other (Figure 1(a)). Elucidating the mechanism underlying this interaction is highly challenging due to their coevolution nature. In our study, we first split this problem into two branches separately (Figure 1(a)). The branch I (Figure 1(a), left) is to study how structure influences dynamics. Specifically, we aimed to understand how synaptic weight affects circuit properties such as firing rate, synchrony, and oscillations, among others, under different synaptic time constants and strengths of background inputs. The branch II (Figure 1(a), right) is the contrary, where we aimed to elucidate how diverse circuit properties would affect synaptic weights through plasticity, while the actual weight is not changed. Finally, the two branches were considered together in self-organized circuit to study the coevolution of network dynamics and structure simultaneously in the presence of plasticity.

The dynamics of neural circuits studied here can perform differently in terms of firing rate and degree of synchrony, consistent with previous studies [26, 33–37]. The key parameters governing these properties are the synaptic coupling strength and the receptor time constants induced by different neurotransmitters. By studying the effect of different artifact spike train in shaping the structure of virtual networks (branch II), we found that the firing rate and synchronous properties of the spike train play a major role. We found that network synchrony is beneficial while the bursting spike of neurons is detrimental to synaptic potentiation. Moreover, gamma oscillation is always accompanied by synaptic potentiation during learning in circuits with plasticity where the dynamics and structure coevolve. Furthermore, we showed that the synchronous dynamics forms a special network structure after learning. E-I balanced inputs received by neurons are temporally broken during learning. In all, the study here showed that gamma oscillation in E-I balanced neural circuits has a beneficial role for effective learning through STDP.

## 2. Material and Methods

### 2.1. Neural Circuit Model

#### 2.1.1. Network and Circuit Dynamics

Our model circuit is composed of 2400 neurons, with 2000 excitatory (*E*), *n*_*E*_, and 400 inhibitory (*I*), *n*_*I*_, neurons. Neurons are connected randomly with directed synapses with probability *p* = 0.2. Each neuron receives background input from 400 independent Poisson trains of rate *f*_background_ = 2.5Hz. Neural dynamics in the network is modeled by conductance-based integrate-and-fire neurons [34] as

(1) $$\begin{array}{c} \tau^{k}\frac{{dV}_{i}^{k}}{dt}=V_{L}-V_{i}^{k}+G_{i}^{kE}\left(t\right)\left(E^{E}-V_{i}^{k}\right)+G_{i}^{kI}\left(t\right)\left(E^{I}-V_{i}^{k}\right),\\ G_{i}^{kI}\left(t\right)=\tau^{k}\underset{j\in \partial_{i}^{I}}{\sum }\underset{n}{\sum }w_{ij}S^{I}\left(t-t_{j}^{n}\right),\\ G_{i}^{kE}\left(t\right)=\tau^{k}\left[\underset{j\in \partial_{i}^{O}}{\sum }\underset{n}{\sum }g^{kO}S^{E}\left(t-t_{j}^{n}\right)+\underset{j\in \partial_{i}^{E}}{\sum }\underset{n}{\sum }w_{ij}u_{j}\left(t\right)x_{j}\left(t\right)S^{E}\left(t-t_{j}^{n}\right)\right]. \end{array}$$

Here, *k* = *E* or *I* denotes the neuron type, and *∂*_*i*_^*k*^ indicates the *k* neighbors of *i* neuron. *V*_*i*_^*k*^(*t*) is membrane potential of neuron *i*, and *t*_*i*_^*n*^ is the *n*th spike of neuron *i*. *w*_*ij*_ is the synaptic strength from presynaptic neuron *j* to postsynaptic neuron *i*, and their initial values are set as

(2) $$\begin{array}{c} w_{ij}=\left\{\begin{array}{c} g^{EE},i\in E,j\in \text{E}\\ g^{EI},i\in E,j\in \text{I}\\ g^{IE},i\in I,j\in \text{E}\\ g^{II},i\in I,j\in \text{I} \end{array}.\right. \end{array}$$

During the simulation, *g*^*EE*^ changes according to the plasticity rules illustrated below. The synaptic time course is described by a biexponential function with two characteristic time constants, the rising time constant *τ*_*r*_^*k*^ and decay time constant *τ*_*d*_^*k*^, both depending on the type of presynaptic neurons. It takes the form

(3) $$\begin{array}{c} S^{k}\left(t\right)=\frac{\Theta \left(t-\tau_{l}\right)}{\tau_{d}^{k}-\tau_{r}^{k}}\left(e^{-\frac{t-\tau_{l}}{\tau_{d}^{k}}}-e^{-\frac{t-\tau_{l}}{\tau_{r}^{k}}}\right), \end{array}$$

where Θ(*t*) is the Heaviside function. We used excitatory synaptic time constant ranging from *τ*_*d*_^*E*^ = 3 ~ 90ms. It is biologically realistic because AMPA receptor has a smaller synaptic time constant at about 5 ms [38] and NMDA receptor has a large synaptic time constant at around 100 ms [39]. Thus, an effective combination of AMPA and NMDA receptors gives the excitatory synaptic time constant lying in the range we studied in this work, and the value of *τ*_*d*_^*E*^ is biologically determined by the ratio between these two receptors. Furthermore, we used *τ*_d_^*I*^ = 10ms, approximately the time constant of a major inhibitory receptor, the GABAa [40]. Different shapes of synaptic current (Eq. (3)) are shown in Figure 1(b).

#### 2.1.2. Short-Term Synaptic Plasticity

We adopt presynaptic short-term plasticity (STP) in the model, which represents the dynamics of neurotransmitter [41]. It is described by two variables, the amount of neurotransmitter *x*_*i*_and its release probability per spike  *u*_*i*_, which are associated with their time constants *τ*_*D*_, the depression time constant, and *τ*_*F*_, the facilitation time constant. In general, *τ*_*F*_ ≫ *τ*_*D*_ holds. These variables are governed by the following equations:

(4) $$\begin{array}{c} \frac{{du}_{i}\left(t\right)}{dt}=\frac{U-u_{i}\left(t\right)}{\tau_{F}}-U\left(1-u_{i}\left(t\right)\right)S_{i}\left(t\right),\\ \frac{{dx}_{i}\left(t\right)}{dt}=\frac{1-x_{i}\left(t\right)}{\tau_{d}}-u_{i}\left(t\right)x_{i}\left(t\right)S_{i}\left(t\right), \end{array}$$

where *δ*(*t*) is the Dirac delta function and *S*_*i*_(*t*) = ∑_*n*_*δ*(*t* − *t*_*i*_^*n*^) is the spike train of neuron *i*. According to the firing history of neurons, short-term potentiation or depression [41–44] can be induced by the change (accumulation and release) of neurotransmitter *x*_*i*_ and the change of release probability *u*_*i*_ , which in turn temporal change the synaptic efficacy.

#### 2.1.3. Plasticity Rule

This synaptic weight changes according to the integrative plasticity mechanism, modeled by a triplet spike-timing-dependent plasticity (STDP), plus a heterosynaptic plasticity and a transmitter-induced plasticity [19]. In our model, the plasticity rule is applied to all E to E synapses in the recurrent network. That is, for two excitatory neurons *i*, *j*, their synapse evolves according to

(5) $$\begin{array}{c} \frac{dw_{ij}}{dt}=\underset{\text{Triplet STDP}}{\underset{⏟}{S_{i}\left(t\right)∙\left(Az_{j}z_{i}^{\text{slow}}\right)-S_{j}\left(t\right)∙\left(Bz_{i}\right)}}-\underset{\text{Heterosynaptic plasticity}}{\underset{⏟}{S_{i}\left(t\right)∙\beta z_{i}^{3}\left(w_{ij}-\tilde{w}\right)}}+\underset{\begin{array}{c} \text{Transmitter}\\ \text{induced plasticity} \end{array}}{\underset{⏟}{\delta_{1}\;S_{j}\left(t\right)}}, \end{array}$$

(6) $$\begin{array}{c} \frac{{dz}_{i}}{dt}=-\frac{z_{i}}{\tau_{\text{STDP}}}+S_{i}\left(t\right), \end{array}$$

(7) $$\begin{array}{c} \frac{{dz}_{i}^{\text{slow}}}{dt}=-\frac{z_{i}^{\text{slow}}}{\tau_{\text{STDP}\_\text{slow}}}+S_{i}\left(t\right) \end{array}$$

In simulation, we set a lower bound of each synaptic weight as 0.001 to prevent negative and zero value.

The plasticity modifies synaptic weight according to presynaptic and postsynaptic traces. Each neuron *i* is accompanied by two synaptic traces, the fast synaptic trace *z*_*i*_ and the slow synaptic trace *z*_*i*_^slow^. They increase by a unit when the neuron has a spike and decays to zero with fast and slow time constants, i.e., *τ*_STDP_, *τ*_STDP_slow_, respectively. Apart from the commonly used fast synaptic trace, we also adopt a slow synaptic trace, which is first introduced in [21]. Slow synaptic trace gives rise to the spiking frequency dependence of plasticity, and it can better match with experimental result [19, 21]. The first term in the right-hand side (RHS) of Eq. (5) is the triplet STDP that potentiates synapse by an amount of *Az*_*j*_*z*_*i*_^slow^ every time a postsynaptic neuron fires and depresses synapse by an amount of *Bz*_*i*_ every time a presynaptic neuron fires, where *A* and *B* are long-term potentiation and depression rates, respectively. In general, considering the effect of a spike in presynaptic neuron at time *t*_pre_^sp^ and a spike in postsynaptic neuron at time *t*_post_^sp^, the synapse linking these two neurons are potentiated when *t*_post_^sp^ − *t*_pre_^sp^ > 0 and depressed when this difference is negative. Imposing a triplet STDP alone is in general unstable in synapse evolution. This is because, for STDP between excitatory neurons, potentiated (depressed) synapses tend to induce more (less) spiking of the postsynaptic neurons, which in turn potentiates (depress) this synapse. Such a positive feedback effect makes the synapse weights diverge. A solution to stabilize triplet STDP is to let it works with other plasticity rules together. The general rule that can stabilize STDP was introduced in [45, 46]. Zenke et al. [19] used heterosynaptic plasticity, described by the second term on the RHS of Eq.(5), to stabilize triplet STDP. Every time when a postsynaptic neuron spikes, the heterosynaptic plasticity reduces the synaptic strength by an amount of$\;\beta z_{i}^{3}\left(w_{ij}-\tilde{w}\right)$, with *β* being a learning rate and $\tilde{w}$ being the target synaptic weight value. The heterosynaptic plasticity rule was found in experiment [24, 25], which suggests that it works in high spiking frequency domain; therefore, a cubic exponent for postsynaptic trace, *z*_*i*_^3^, is used. Transmitter-induced plasticity, the third term in the RHS of Eq. (5), prevents neurons from silence. It may represent spine growth [47] or a kind of long-term potentiation to counteract Hebbian long-term depression [48]. Its effect is not crucial for our study here, since we study relatively short simulation time ~100 s.

#### 2.1.4. Learning and Memory Encoding

We encoded the memory into the network during learning by adding stimulus to a subset of neurons in addition to background input, which facilitates the formation of a cluster structurally by these neurons. Unless otherwise specified, at *t* = 30s, an extra stimulation input representing a learning signal is applied to 200 chosen excitatory neurons to model the coding of a single memory. For those chosen neurons, their background rate is increased from *f*_background_ to *f*_stim_ = *As* × *f*_background_, where *As* is an augment factor. This extra stimulation input lasts until *t* = 100s, and the background rate of the chosen neurons is tuned to 0.5*f*_background_ lasting for 2 s to remove persistent activity [41, 49]. After that, their background input is tuned back to the default rate *f*_background_ and the simulation is ended at *t* = 110s.

The biological meaning and value of parameters in our model is summarized in Table 1.

### 2.2. Statistical Index and Analysis

#### 2.2.1. Spike Count Series

For analyzing neural dynamics, we first constructed the neuron spike train series as follows. The time axis is first divided into consecutive time windows with sizes Δ*t* ms. The number of spikes of neuron *i* are then counted in each window to obtain a discrete sequence *N*_*i*_(*t*), which is designated as the spike count series of neuron *i* with time window size Δ*t*.

Furthermore, we defined a binary spiking series *B*_*i*_(*t*) = 0, if *N*_*i*_(*t*) = 0 and *B*_*i*_(*t*) = 1, if *N*_*i*_(*t*) > 0 for each neuron.

The number of spikes of the whole neuron population can be counted in each window. This constructs the population spike count series *N*_*α*_(*t*) for the E and I populations, respectively. Furthermore, *q*_*α*_(*t*) = *N*_*α*_(*t*)/*n*_*α*_Δ*t* is the population averaged firing rate series.

#### 2.2.2. Synchrony Index

We used a synchrony index to quantify the synchronized spiking of neurons in the circuit [34] as

(8) $$\begin{array}{c} SI_{ij}=\frac{\sum_{t}B_{i}\left(t\right)B_{j}\left(t\right)}{\sqrt{\sum_{t}B_{i}\left(t\right)\sum_{t}B_{j}\left(t\right)\;}}, \end{array}$$

where *B*_*i*_(*t*) is the binary spike series binned with Δ*t* = 3ms. If the two neurons *i*, *j* are completely synchronous, then *SI*_*ij*_ = 1, and if they are completely asynchronous, *SI*_*ij*_ = 0.

#### 2.2.3. Gamma Power

The power spectrum density of network oscillation is calculated by Fourier transform of the mean-detrended population firing rate *q*_*α*_(*t*) constructed with Δ*t* = 600ms. We define the gamma power as the integration of the power spectrum density from 28 Hz to 40 Hz [27, 28].

In circuits with plasticity, high firing neurons within the 200 coding neurons, which are defined here as neurons having spike in 95% of bins in the firing rate series with width Δ*t* = 50ms, are picked for calculating frequency, firing rate, synchrony, and oscillation. This is because we are interested in the properties related to plasticity and the synaptic potentiation/depression are concentrated at synapses of high firing neurons (synapses of low firing neurons only change slightly). No significant conclusion can be drawn if we take all the neurons into account.

## 3. Results

### 3.1. Circuit Dynamics without Plasticity

We considered a randomly connected conductance-based excitatory-inhibitory neuronal circuit [34] together with triplet STDP, heterosynaptic plasticity [19], and transmitter-induced plasticity. We studied the process of memory encoding with learning. Details of the model are presented in Methods. In this section, we investigated the circuit dynamical properties without plasticity mechanism. The analysis in this subsection is carried out for the 20 neurons with the highest firing rates.

**Figure 2:**
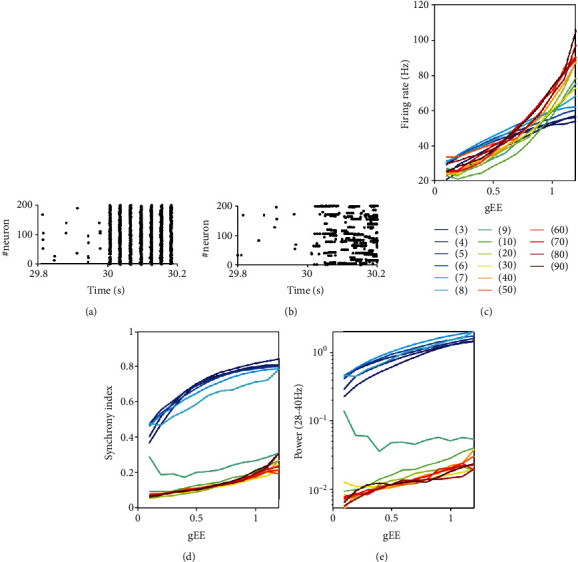
Dynamical properties of the circuits without plasticity. (a, b) Raster plot of the spiking time of 200 stimulated neurons (extra stimulation onset at = 30s for *g*^*EE*^ = 0.1. (a) Highly synchronous state with *τ*_*d*_^*E*^ = 6ms. (b) Asynchronous state, *τ*_*d*_^*E*^ = 90ms. (c–e) Circuit properties with respect to synaptic strength during extra stimulus. The cases of different *τ*_*d*_^*E*^ values are plotted in different colors, and the *τ*_*d*_^*E*^ values corresponding to different colors are shown in the bar. (c) Network firing rate. (d) Synchrony index. (e) Gamma power. Other parameters are set as *τ*_*d*_^*I*^ = 8ms, *As* = 2.5.

First, we studied the effect of different excitatory synaptic time constants (*τ*_*d*_^*E*^) and the excitatory synapse weight *g*^*EE*^ on the network dynamics. Before the extra learning stimulation (at *t* = 30s), the circuits with different *τ*_*d*_^*E*^ are in low firing rate, low synchrony, and without apparent network oscillations (Figures 2(a) and 2(b)). After applying the extra stimulation, the dynamic feature of circuit becomes apparent, and circuits with smaller *τ*_*d*_^*E*^ tend to support synchronous spiking (Figures 2(a) and 2(b)).

Second, we explored how synaptic weight affects the dynamics regarding synchrony, oscillation, and firing rate. Generally, smaller coupling strength *g*^*EE*^ or smaller excitatory synaptic time constant *τ*_*d*_^*E*^ induces lower firing rates (Figure 2(c)). The synchrony index (Figure 2(d)) and gamma power (Figure 2(e)) (details in Methods) have similar dependence on synaptic strength. They increase as *g*^*EE*^ increases or *τ*_*d*_^*E*^ decreases. Interestingly, there is a sharp change in these dependence relations when  *τ*_*d*_^*E*^ is close to  *τ*_*d*_^*I*^, which inspires us to roughly distinguish two network states as follows. When  *τ*_*d*_^*E*^ is smaller than  *τ*_*d*_^*I*^, the synchrony and gamma power are relatively strong. In this case, we termed the dynamics under stimulation input as synchronous dynamic state. In contrast, when  *τ*_*d*_^*E*^ is larger than  *τ*_*d*_^*I*^, synchrony and gamma power are weak, and we termed the dynamics under stimulation input as asynchronous dynamic state. Finally, we will also explore the dynamic states with *moderate synchrony* when  *τ*_*d*_^*E*^ is close to  *τ*_*d*_^*I*^. These definitions apply to the following study of networks with or without plasticity.

### 3.2. The Effect of Spiking Dynamics on Plasticity

In neural circuits with plasticity mechanism, the synaptic weights are shaped by network dynamic properties, especially, the degree of synchrony and firing rates. To understand the effect of network spiking dynamics on plasticity, we first studied the neural circuit without plasticity and with extra stimulation starting at *t* = 30s. Next, the plasticity rule is applied to artifact spike trains generated by manipulations of the spike trains of 200 coding excitatory neurons obtained from this network simulation. Then, we imposed the plasticity rule with these artifact spike trains on a virtual network with the same topology connection as in the original circuit, to see how the synaptic weights can be changed. Note that here we did not consider how this change of synaptic weights in turn shapes the network dynamics. In this way, we separated the coevolution of dynamics and synapses and only considered the impact of dynamics properties on the evolution of synapses under plasticity (Figure 1(a), right).

We aimed to explore how the change of (virtual) synaptic strength depends on the firing rate and synchrony index of the spiking series with the following approaches. (1) To examine the effect of different degree of synchronization, we randomized a portion of spike time within every 100 ms interval. This can generate spike trains with different synchrony index while almost keeping the average firing rate (in a short time scale of 100 ms) of neurons. (2) To investigate the effect of different average firing rates, we insert empty bins with a certain length *T*_*em*_ into the spike trains every 100 ms. This can generate spike trains with different firing rate (in a short time scale) while keeping the synchrony index. Note that this manipulation changes the time ranges of the spike trains. (3) We also tried the combination of the above two schemes (i.e., inserting empty bins together with randomizing some spikes) that simultaneously changes the firing rate and synchrony index.

**Figure 3:**
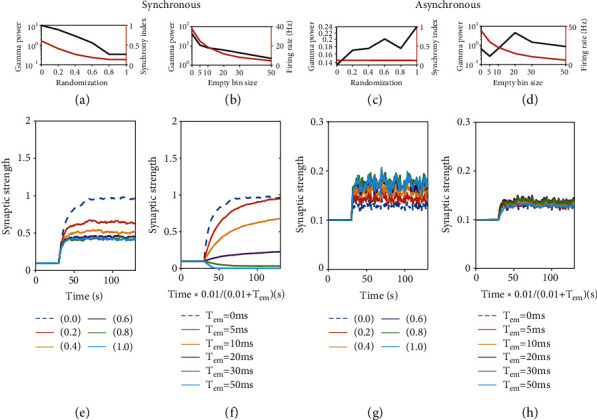
Effect of firing rate and synchrony on virtual synaptic weight evolution under plasticity. (a, e, c, g) The effect of spike time randomization. (b, f, d, h) The effect of empty bin inserting. (a–d) The gamma power and firing rate of the network depend on the proportion of randomization/size of empty bin insert. (e–h) The change of synaptic weight with time. The time axis in (f, h) is modified based on the empty bins insert. (a, b, e, f) Synchronous state. *τ*_*d*_^*E*^ = 6ms. (c, d, g, h) Asynchronous state. *τ*_*d*_^*E*^ = 90ms. The other parameters are set as *τ*_*d*_^*I*^ = 8ms, *As* = 3.5.

When applying plasticity to the spike trains from highly synchronous dynamics in the presence of extra stimulations (small *τ*_*d*_^*E*^), the synaptic weight quickly increases and stabilizes to a large value (Figure 3(e), dashed line). When spikes are randomized to decrease the synchrony, the final average stabilized synaptic weights, the gamma power, and the time required for stabilizing the synaptic weight get smaller (Figure 3(a)). Next, by inserting empty bins, we found that a lower firing rate would produce lower finalized synaptic weight (Figure 3(f)), lower gamma oscillation power (Figure 3(b)), and longer stabilizing time. Moreover, if the firing rate is low enough, the role of synaptic plasticity changes from potentiation to depression (Figure 3(f)).

In the asynchronous dynamics, neurons often fire in a burst way (Figure 2(b)), that is, a neuron can have several spikes in a very short time period (see distribution of instantaneous rate in Figure S1 in the Supplementary Material where in the asynchronous dynamics, there is a peak at high firing rate up to 200 to 300 Hz). In the asynchronous state, the spike time randomization does not change the synchrony index (Figure 3(c)) since the correlation is already very low, but it does change the synaptic weight evolution. Different from the case of synchronous dynamics, here, the synaptic strength increases when a larger portion of spikes is randomized (Figure 3(g)). This is because the high instantaneous firing rate due to neuron bursts can strengthen the heterosynaptic plasticity (the *z*_*i*_^3^ term in Eq. (5) will become very large) that suppresses synaptic potentiation, and bursts are destroyed by spike time randomization. Furthermore, the final stable synaptic strength increases only slightly with the reduction of the firing rate by inserting empty bins (Figure 3(h)). This may be because the effect of inserting empty bins for destroying bursts is not as strong as randomization.

By combination of the randomization of spike times and the insert of empty bin, spike trains with different combinations of firing rate and synchrony index can be generated. We found that for synchronous dynamics, the final stable synaptic weight depends mainly on firing rate but slightly on synchrony index (See Figure S2C in the Supplementary Material), since a too high instantaneous firing rate would activate heterosynaptic plasticity and a too low firing rate would favor triplet STDP depression. Thus, the synaptic weight potentiation requires a suitable range of firing rate. Besides, synchrony index and gamma power are accompanied on synaptic potentiation (Figures 3(a) and 3(b)). However, for asynchronous dynamics, synaptic weight can only potentiate slightly (See Figure S2A in the Supplementary Material) due to the burst spiking nature of the neurons.

### 3.3. Circuit with Plasticity

In the following, we study the interplay between structure and dynamic properties as a whole in the E-I circuit with plasticity, where the synaptic weights and dynamics patterns self-organize through coevolution. We referred the results in section 3.2, where the spiking dynamic is not influenced by the updated network structure, to as the manipulation results.

**Figure 4:**
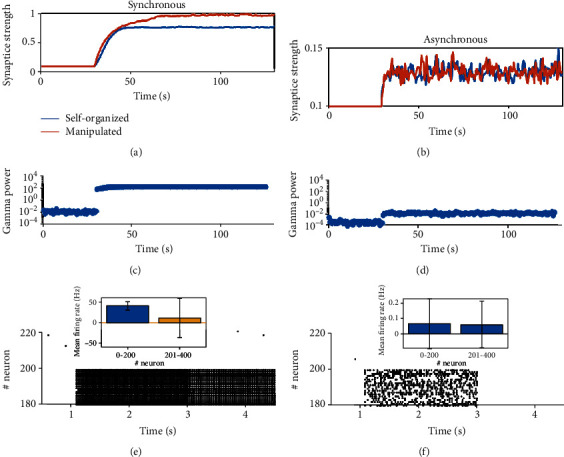
Properties of circuits with plasticity. (a, b) Synaptic weight evolution. Blue curves are results from networks with plasticity, whereas orange curves are from virtual networks in Figure 3. (c, d) The evolution of gamma oscillation and synaptic weight in different dynamical state. (e, f) The ability of working memory maintenance after recalling in the network after learning. Extra stimuli (*As* = 5) are applied to the neuron group with memory encoded for 2 s (from 1 s to 3 s). The persistent firing ability of the group after extra stimuli removal is checked. The insets compare the firing rate of the memory-coding group and the firing rate of other neurons during the persistent period. (a, c, e) Synchronous state. *τ*_*d*_^*E*^ = 6ms. (b, d, f) Asynchronous state. *τ*_*d*_^*E*^ = 90ms. The other parameters are set as *τ*_*d*_^*I*^ = 8ms, *As* = 3.5.

First, we examined the synaptic weight evolution. In the case of synchronous dynamics, the coevolution dynamics induce a slightly lower final stable average synaptic weight (Figure 4(a)) compared with the manipulation results. This is because, under coevolution dynamics, neurons can be more excited after the potentiation of the synapses, producing a higher firing rate that supports stronger heterosynaptic plasticity to reduce the potentiation. However, the synaptic weight evolution under asynchronous dynamics does not show much difference (Figure 4(b)) compared with manipulation results.

Below, we investigated how gamma power evolves in the self-organized plastic circuit. We calculated the gamma power (see Method) from the population spike train of high firing neurons from simulation result for every short period with a length of 600 ms. The initial gamma power is very weak (around 10^−3^ to 10^−2^) with weak background inputs, and it undergoes a jump (Figures 4(c) and 4(d)) when extra stimulus starts. In circuits with synchronous dynamics, the synaptic weight potentiates and gamma oscillation increases significantly together (Figure 4(c)). In circuits with asynchronous dynamics, the increases of gamma oscillation and the synaptic weight are both tiny (Figure 4(d)).

We further tested the performance of supporting working memory of the network after learning under different dynamics modes. Under synchronous dynamics, the significant potentiation of synaptic weights facilitates the maintenance of working memory (Figure 4(e)) after initial recall, whereas for asynchronous dynamics, the network after learning is not potentiated enough for the robust maintenance of working memory (Figure 4(f)).

**Figure 5:**
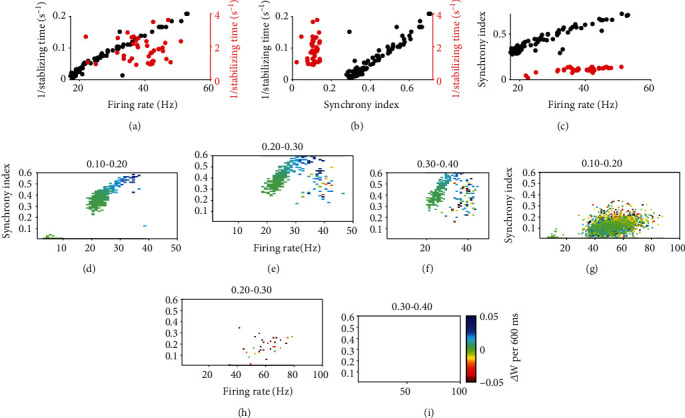
The process of synaptic stabilizing in circuits with plasticity. (a) The relation between stabilizing time and firing rate. (b) The relation between stabilizing time and synchrony index. (c) The relation between firing rate and synchrony index. In (a–c), black/red dots are results under synchronous/asynchronous dynamics. (d–i) The relationship between the change of synaptic weight with respect to the instantaneous firing rate and synchrony index during the simulation. The studied ranges of synaptic weight are shown as title above the subfigures. (d–f) Under synchronous dynamics with *τ*_*d*_^*E*^ = 3, 4, 5 ⋯ 9ms. (g–i) Under asynchronous dynamics with *τ*_*d*_^*E*^ = 20, 30, 40 ⋯ 90ms. Other parameters are*As* = 2, 2.5, 3, 3.5. and *τ*_*d*_^*I*^ = 8ms.

Furthermore, we were curious about how fast the synaptic weight can be stabilized, which reflects the learning speed. We explored the stabilizing times (defined as the time required for reaching half maximum synaptic weight) in circuits with plasticity under different initial dynamics states (at the time right after extra stimulation onset) such as different firing rates and synchrony using both different *τ*_d_^*E*^ and *As*. Under synchronous dynamics, it is found that stabilizing time in circuits with plasticity is inversely proportional to the initial firing rate (Figure 5(a)), as well as to the initial synchrony index (Figure 5(b)). There is a linear relationship between the initial firing rate and synchrony index (Figure 5(c)). Thus, the increase of firing rate is important to stabilize the synapses quickly. As the stabilized synaptic weights are different for synchronous and asynchronous state, we further studied the speed of synaptic potentiation with respect to firing rata and synchrony, and we recorded the synaptic weight change in different synaptic weight intervals (shown above Figures 5(d)–5(i)). A higher initial firing rate produces a higher initial synaptic weight change (Figure 5(d)). Potentiated synapses generate higher firing rate and in turn trigger stronger heterosynaptic plasticity and thus more depression (Figures 5(e) and 5(f)). Consequently, the stabilized synaptic weight would be less (Figure 4(a)) but also takes less time to stabilize. In contrast, asynchronous state stabilizes quickly (<600 ms), and the synapses are only potentiated slightly (Figure 4(b)). The higher initial firing rate due to bursting in the asynchronous state (Figure 5(g)) causes strong heterosynaptic plasticity to prevent the synapses from further potentiation (Figures 5(h) and 5(i)). However, the speed of synaptic potentiation, that is, the amount of potentiation in unit time, is higher under synchronous dynamics (Figures 5(d) and 5(g)).

**Figure 6:**
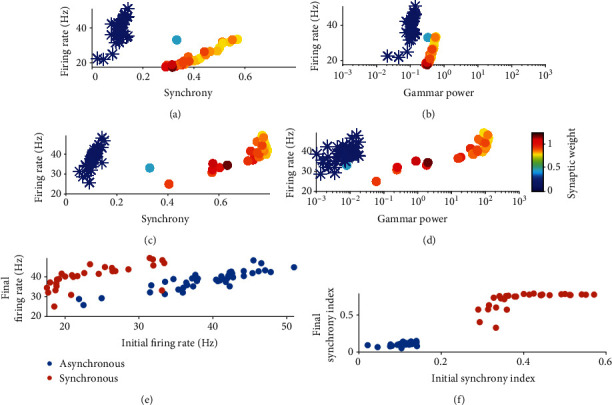
The relationship between synaptic potentiation and network dynamics in circuit with plasticity. (a, b) The relationship between the final stable synaptic weight (represented by color) and initial firing rates and gamma power. (c, d) The relationship between the final stable synaptic weight and final firing rates and gamma power. In general, there is a positive correlation between the gamma power/synchrony index and the stable synaptic weight. Stars represent the results in asynchronous states whereas dots represent results in synchronous and moderate synchronous states. (e, f) The relationship between initial and final firing rate and synchrony index, respectively. In (a–f), parameters used are *τ*_*d*_^*E*^ = 3, 4, 5 ⋯ 10,20,30 ⋯ 90ms, *τ*_*d*_^*I*^ = 8ms, *As* = 2, 2.5, 3, 3.5.

In circuits without plasticity, it was found that the increase of synaptic weight results in increased firing rate (Figure 2(c)). This relation does not hold in the presence of plasticity, as too high firing rate would induce synaptic weight depression due to heterosynaptic plasticity. First, the initial firing rate and gamma power of the network (in the beginning stage of extra stimulus onset, Figures 6(a) and 6(b)) is positively correlated to those after learning (Figures 6(e) and 6(f)). In general, we found that high synchrony index and gamma power facilitates the increase of synaptic weight (Figures 6(a)–6(d)), whereas the effect of firing rate is not pronounced relatively. It suggests that synchrony and gamma oscillation are beneficial for synaptic potentiation in the plastic E-I networks.

The above studies suggest that there should be essential differences in the network structures after learning under different dynamic states. To investigate the essential features about this structure changes, we performed various shuffling of the connectivity matrix of the subgroup of excitatory neurons for memory encoding adopted from the plastic circuits when they have evolved into the stable state. Then, we simulated the whole network dynamics without plasticity after shuffling this sub connectivity matrix with stimulation input applying at 30 s. In particular, we focused on exploring whether the structure can still support synchronous dynamics after shuffling.

**Figure 7:**
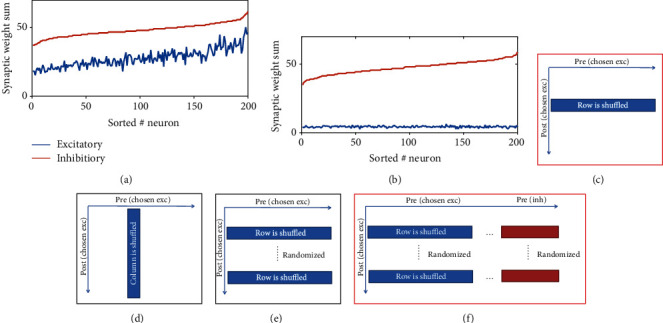
Special circuit structures formed by plasticity. (a, b) The relation between the sums of incoming excitatory and inhibitory synaptic weight of different neurons in the plastic circuit after learning, sorted according to the ascending order of inhibitory weight sum. Parameters are set as *τ*_*d*_^*I*^ = 8ms, *As* = 3.5. (a) Synchronous with *τ*_*d*_^*E*^ = 6ms. (b) Asynchronous with *τ*_*d*_^*E*^ = 90ms. (c–f) Illustration of shuffling schemes. Each figure represents a connectivity matrix. In (c–e), we shuffled the elements of the connectivity sub-matrix of the chosen excitatory neurons (to encode the memory). (c) The input elements of each neuron (each row) are shuffled (total input is preserved). (d) The output elements of each neuron (each column) are shuffled (total input is changed). (e) The elements of each row are first shuffled and then the row is randomly shuffled (total excitatory input is preserved, but total inhibitory input is changed). The shuffling scheme of (f) the elements of each row of the submatrix of excitatory neurons is first shuffled, and then, the row of the entire matrix is randomly shuffled (total excitatory and inhibitory input are preserved). If the shuffled matrix results in synchronous/asynchronous dynamics, then it is marked in a red/black box.

The major finding is that the network can preserve synchronous dynamics when the incoming connections of the memory-coding neurons are shuffled (total input is preserved) (Figure 7(c)). On the contrary, the network cannot preserve synchronous dynamics when the outgoing connections of the neurons are shuffled (total input is changed) (Figure 7(d)). If the row of the submatrix is further randomized (rows are moved as a whole) after shuffling the incoming connections, synchronous dynamic is destroyed (Figure 7(e)). However, synchronous dynamics can be preserved if the randomization of row is performed in the whole connectivity matrix (Figure 7(f)), that is, preserving the inhibitory inputs in this randomization. Taken together, it implies that the ability for network supporting synchronization is related to the sum of incoming synaptic weights.

Interestingly, we found that there is a high correlation among the sum of excitatory and the sum of inhibitory synaptic weights in the learning-stabilized matrix under synchronous dynamics (Figure 7(a)), suggesting that neurons receiving strong inhibitory synapses (these inhibitory synapses are without plasticity) also receive stronger excitatory synapses (these synapses are with plasticity). This special network structure is not present under asynchronous dynamics (Figure 7(b)). To further confirm networks with such structural features really favors synchronous spiking dynamics, a random circuit with similar synaptic weight sum distribution is generated and we found that the generated circuit indeed supports synchrony dynamics (See Figure S3C in the Supplementary Material).

Synapses potentiation leads to burst firing when excitation is strongly over inhibition (see Figure S3A in the Supplementary Material). When a neuron gets burst, its synapses would be suppressed by heterosynaptic plasticity. Therefore, neuron stays synchrony with only a few bursting events. As neurons receiving stronger inhibitory current need more excitation for burst firing to happen, their synapses can potentiate more before the heterosynaptic plasticity sets in. Hence, the correlation between excitatory and inhibitory inputs is developed. We should note that it is not related with the number of incoming excitatory connections but the sum of the incoming excitatory weights.

### 3.4. The Moderate Synchronous Dynamics

**Figure 8:**
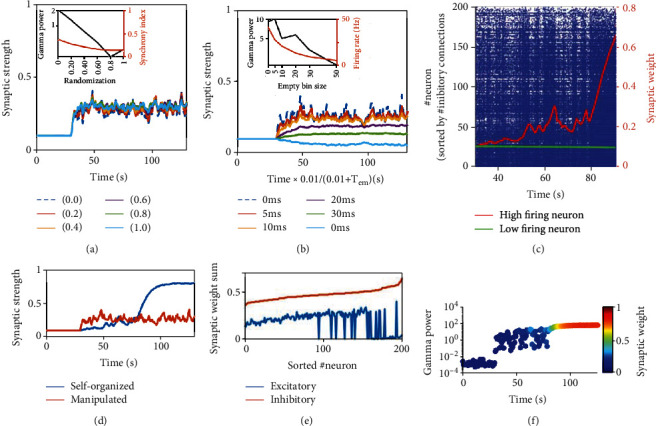
The dynamic properties of learning under moderate synchronous state. (a, b) Effect of firing rate and synchrony on synaptic weight evolution under plasticity with spike manipulation. (a) The effect of spike time randomization. (b) The effect of empty bin inserting. (c) Raster plot. Neuron number is sorted in an increasing order of inhibitory input connections. Red curve is the mean excitatory synaptic strength of high firing neurons, which increases during synchronous windows and decreases during asynchronous windows. Green curve is the mean excitatory synaptic strength of low firing neurons. (d) The synaptic weight evolution. (e) Sum of incoming excitatory and inhibitory synaptic weight of neurons in the plastic circuit after learning, sorted according to the ascending of inhibitory sum. (f) The evolution of gamma oscillation and synaptic weight in moderately synchronous states.

From the comparison between synchronous and asynchronous states, we have seen that the effect of learning depends significantly on the background dynamics. This raises a question: whether there are special properties on the boundary between these two dynamics regimes, that is, the moderate synchronous state. We found that under moderate synchronous dynamics, the transient network activity can switch between asynchronous burst and weak synchronization. Therefore, such a switching state should have both features in synchronous and asynchronous states. In static networks without plasticity, this switching dynamic is a long-lasting property (data not shown here). In networks with plasticity, this switching state is only temporal and the network will finally evolve into synchronous dynamics (Figure 8(c)).

We first tested the effect of manipulation spike trains from circuit of moderate synchronization on shaping a virtual network structure with similar methods in Figure 3. For spike time randomization, the stable synaptic weights are almost unchanged with synchrony index (Figure 8(a)). When inserting an empty bin to reduce the firing rate, the stable synaptic weight is first kept unchanged and then starts to reduce and even becomes depressed (Figure 8(b)) when the firing rate is lower than a level. Next, we studied the network with plasticity under moderate synchronous dynamics. Interestingly, the transient synaptic weight evolution in plastic network differs strongly compared with manipulation results (Figure 8(d)). The synaptic weight increases during temporally synchronous periods and recovers during asynchronous periods, but this switching period is transient. The circuit will finally evolve into a fully synchronous state (e.g., from t = 80s in Figure 8(c)), and the synaptic weight increases after that until reaching the maximum (Figure 8(d)). The gamma oscillation increases gradually during the time period with switching synchronous and asynchronous spiking (Figure 8(f)), where the gamma oscillation power and synaptic weight have large fluctuations. Once the circuit reaches the synchronous state, both synaptic weights and gamma oscillation power increase to the stable value quickly. Neurons in moderately synchronous circuit can perform differently with respect to the amount of inhibitory input they receive, as shown in Figure 8(c) (neurons sorted according to total inhibitory synaptic weights). The evolution of the mean excitatory synaptic weight is also plotted in Figure 8(c). For neurons receiving too many inhibitory inputs, they do not have enough instantaneous excitatory current to overcome inhibitory suppression and they fire sparsely. In contrast, neurons receiving relatively fewer inhibitory inputs can overcome the inhibitory suppression and have the potential to perform like synchronous state, with a similar mechanism described above for the synchronous state.

A special relationship between excitatory and inhibitory current causes moderately synchronous state to switch between synchronous and asynchronous because its excitatory current is both high in amplitude (not as high as synchronous state) and long lasting (not as long as asynchronous state), as *τ*_*d*_^*E*^ is in between that of synchronous and asynchronous state. Once neurons are synchronized, inhibitory neurons can be activated by synchronized excitatory current to induce strong inhibition and terminate the synchrony event. Therefore, the circuit becomes asynchronous again and neurons which receive few inhibitory inputs may start to burst, and this process underlies the temporal switching between synchronous and asynchronous states. When plasticity is applied, synapses are potentiated during synchrony and depressed during asynchrony (Figure 8(c)). However, when the synapses are potentiated to a large enough value, the excitatory current is too strong that inhibitory current cannot terminate the synchrony anymore and the circuit starts to develop strong synchrony.

### 3.5. Other Dynamic Properties during Learning

**Figure 9:**
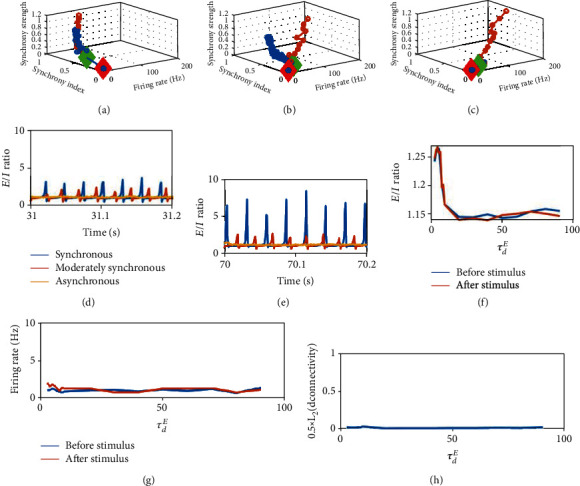
Other dynamic properties. (a–c) Blue curves are the evolution dynamics in circuits with plasticity starts from strength 0.1. Red diamond represents the start of simulation, and green diamond represent the start of extra stimulus. Orange curves are the results of static circuits with different synaptic strengths. (a) Synchronous dynamics with *τ*_*d*_^*E*^ = 6ms. (b) Moderately synchronous dynamics with *τ*_*d*_^*E*^ = 10ms. (c) Asynchronous dynamics with *τ*_*d*_^*E*^ = 90ms. The other parameters: *τ*_*d*_^*I*^ = 8ms, *As* = 3.5. Transient excitatory and inhibitory current ratio in early stage of stimulus (d) (<5 s after applying extra stimulus input at *t* = 30s) and late stage of stimulus (e) (after synaptic weight stabilized). (f) Average E/I ratio before extra stimulus and after the removal of stimulus. (g) Firing rate before and after extra stimulus. (h) L2 norm of the difference of the connectivity matrices (reshaped to 1d and normalized) at the removal of the extra stimulus and 10 s later under normal background input.

We simulated circuits with plasticity under different background dynamics, to study the transient evolution of dynamic properties. Every time, we checked the corresponding dynamic properties of a static circuit sharing the same average synaptic weight but without plasticity, to examine their difference. Here, the synaptic weight, average firing rate, and synchrony index among high firing neurons in every 600 ms periods during the coevolution process are considered and compared with the firing rate and synchrony index among the same number of highest firing neurons in a static circuit with differen*tg*^*EE*^ in the range [0.1, 1.2]. It was found that the coevolution dynamics of synchronous and asynchronous plastic circuits are very close to that of static circuit at different coupling strength *g*^*EE*^ (Figures 9(a) and 9(c)), suggesting that although the network architecture is changed by plasticity, but it does not significantly alter the plastic network dynamics in these two states. However, the moderately synchronous circuit is strongly different from the corresponding static circuit (Figure 9(b)). In the static circuit, it is switching between asynchronous and synchronous state in all synaptic strength tested (0.1-1.2), and the synchrony index is relatively small. However, in the self-organized plastic circuit, it differs from the dynamics of static circuit, and it becomes synchronous monotonically at last (Figure 8(c)). Therefore, it suggests that the plasticity has significantly changed the dynamics.

Biological neural circuits are reported to work in an excitation-inhibition (E-I) balanced condition [50, 51]. Our model can maintain an overall E-I balanced current input (the time-average E/I ratio in our model is close to 1) on average, and this property can maintain after applying the strong extra stimulus. Asynchronous dynamics can best support E-I balanced input (Figures 9(d) and 9(e)). Considering the transient balance, for moderate synchronous and synchronous dynamics, there is a temporally strong imbalance corresponding to gamma oscillations during learning (Figures 9(d) and 9(e)). It suggests that the temporal imbalance is beneficial to effective learning.

Next, we examined whether the network is stable, and the balance can be restored when extra stimulus stops after learning. To check whether the network connectivity still changes significantly with plasticity under normal background input, the L2 norm difference between the connectivity (reshaped to 1d and then normalized, with value in [0, 2]) among the chosen neurons for memory encoding right after the removal of extra stimulus input and 10 s later is measured. It was found that the connectivity only changes slightly (Figure 9(h)). Firing rate (Figure 9(g)) and average E/I ratio (Figure 9(f)) are also returned to the levels similar as the circuit before learning with the stimulation.

## 4. Discussion

In this work, we studied the dynamics of plasticity neural circuit under the learning process with the combination of triplet plasticity, heterosynaptic plasticity, and transmitter-induced plasticity, with particular focus on the role of gamma oscillation. To tackle the challenging coevolution of network structure and dynamics, we first separately explored the effect of structure on dynamics and vice versa. We then unified these understandings to elucidate the principles of dynamics state-dependent learning in E-I neural circuit with plasticity.

### 4.1. Summary of the Finding

We first studied the static circuit without plasticity and found that networks with small *τ*_*d*_^*E*^ show higher synchrony. Furthermore, synchrony in network spiking and gamma oscillation power increase together with coupling strength *g*^*EE*^ throughout these cases.

Through studying the effect of spiking dynamics on shaping a virtual network structure through plasticity, we found that firing rate plays a crucial role. Firing rate needs to be in a suitable range in order to induce potentiation. Synaptic potentiation is restricted by heterosynaptic plasticity in the case of too high firing rate and by triplet plasticity rule in the case of too low firing rate. Synchrony index and gamma power show greater value when potentiation happens.

These understandings from the separate studies shed light on the properties of coevolution of dynamics and synaptic weights in plastic circuit. Increasing synchrony (higher gamma power and synchrony index) is always beneficial to synaptic potentiation, but there is no such relation on firing rate. It thus suggests that gamma oscillation may be more important for synaptic potentiation in learning process in biological E-I circuits. Furthermore, the circuit structure after learning depends on the original basic dynamic states (different degrees of synchrony). The effective learning where the synaptic weights are potentiated is accompanied by a temporal deviation from E-I balance to the case of slight dominance in excitation. The E-I balance can be restored after learning when extra stimulus input is removed.

### 4.2. Gamma Oscillation

The origin of gamma oscillation is still under debate in the literature. It is suggested that gamma oscillation is due to inhibitory loops [11]. Another study found that controlling long-range synaptic weight, synchronization between two columns of E-I neural circuits [36] can generate gamma oscillation in the cross-correlograms between the two E-I circuits' population firing rates. It is known that mediated by synaptic kinetics, E-I balanced neural circuits can support such fast oscillation out from low firing rate [26, 34], which is the way to produce gamma oscillation in our study.

Furthermore, it was discovered that long-range axonal delay can modulate oscillation between gamma and beta [52]. In the moderate synchronous state, we observed the switching dynamics between asynchronous spiking and synchronous spiking. If the switching frequency is in theta band, it is highly similar to the spindle [6, 53, 54] observed in the hippocampus during sleeping, which has been assumed to relate to memory consolidation. It is also found in an experimental study that U1 snRNA overexpression mice, having weaker gamma oscillation than normal mice due to reduction in inhibition-related proteins, have deficit in learning [55]. It may be possible to further explore the phenomena related to sharp-wave ripple and its functional benefits in our model.

### 4.3. The Role of Plasticity in Learning

It has been shown that heterosynaptic plasticity can prevent explosive synaptic weight potentiation [24, 25]. However, it is unclear what kind of circuit structure can be formed by heterosynaptic plasticity. Our model uses a combination of three plasticity roles. The triplet STDP is mainly responsible for synaptic potentiation, bounded by the heterosynaptic plasticity rule, while the transmitter-induced plasticity does not play a significant role since our simulation time is not long enough. Thus, the final stable network structure is mainly shaped by heterosynaptic plasticity. We found that heterosynaptic plasticity shapes the circuit into a structure where the sum of excitatory input weights of a neuron is correlated/uncorrelated with the sum of inhibitory input weights of a neuron under the conditions of synchronous/asynchronous background dynamics, respectively.

### 4.4. Learning with Gamma Oscillation in Biological Neural Circuits

Our work using a biologically plausible E-I circuit confirmed several previous studies and provided a new understanding. Cell assembly formation through spike-timing-dependent plasticity has been studied previously [19, 56–59]. It has been shown that during learning, plasticity shapes the circuit synaptic weights and spiking correlation [45, 60–62]. The research of learning in biological neural networks has mainly focused on the recall reliability of working memories and the usage of the network for performing classification tasks, but seldom considered the effect of gamma oscillation in learning process, although gamma oscillations have been widely observed to accompany learning in experiments [6, 7, 11, 27, 28]. Here, we found that the increase of gamma power and synchrony always facilitates potentiation in plasticity circuits, providing the understanding behind the experimental observations. The synchrony during gamma oscillations within suitable firing rate is beneficial to synaptic potentiation, which in turn forms a network structure better support gamma oscillations. Thus, gamma oscillation is important for forming cell assemblies, leading to efficient learning.

We have considered the variation of excitatory synaptic time constant (*τ*_*d*_^*E*^) on the dynamical modes of the circuits. AMPA receptor has a smaller synaptic time constant ~5 ms [38], and NMDA receptor has a large synaptic time constant ~100 ms [39]. Thus, the value of *τ*_*d*_^*E*^ used in the network is biologically determined by the ratio between these two receptors. Experiments show that AMPA and NMDA receptors exist extensively in synapses, and their ratio varies in different brain region [63–65]. For example, the number of NMDA receptor is larger in the prefrontal cortex [63] than in the visual cortex. Thus, the fact that neural circuits in different brain systems favor different *τ*_*d*_^*E*^ sheds light on the differential functional role of different brain systems in learning.

### 4.5. Outlook

In this study, we did not consider inhibitory plasticity, which is mainly thought of as a kind of homeostatic plasticity that works in a longer timescale [19, 35]. It was shown that this kind of plasticity helps to maintain tight E-I balance automatically after learning under a wide range of initial parameters. However, how inhibitory plasticity changes the circuit structure is not yet clear. This deserves further study in the future.

So far, we have considered the encoding and retrieval of a single memory cluster. We have preliminarily tested to encode multiple memory clusters successively whose connections are nonoverlapping with each other in the network and found that the learning processes of different clusters work almost independently with each other (data not shown). This issue needs further exploration in future work. This is consistent with our results that the learned circuit is structurally stable under plasticity with weak background input. An interesting extension is to consider overlapping memories which may interfere with each other and generate more complicated interaction between gamma oscillations within each cluster and their learning process.

How to analytically predict the stable synaptic weights after learning through STDP is a challenging issue. Existing theory [66, 67] tended to use mean-field approximation to analyze the mean firing rate, mean synaptic trace, and their covariance. However, the heterosynaptic plasticity rule used in our model involves a cubic exponent of synaptic trace and thus requires high-order covariance, which induces large errors in the corresponding approximation. An effective theory to analyze the effect of heterosynaptic plasticity is still lacking and deserves further study.

## Figures and Tables

**Table 1:** Parameter used in neural circuit.

Description	Symbol	Value
Numbers of E, I neurons	*N*_*E*_, *N*_*I*_	2000, 400
Network connection probability	*p*	0.2 [26]
Background input connection number	*N*_*O*_	400
Default firing rate of background input (per connection)	*f*_background_	2.5 Hz
Augment factor	*As*	3.5
Axonal delay	*τ*_*l*_	1 ms [26]
Leakage potential	*V*_*L*_	-70 mV [26]
Threshold potential	*V*_th_	-50 mV [26]
Rest potential	*V*_rest_	-60 mV [26]
Membrane time constant for E, I neurons	*τ* ^*E*^, *τ*^*I*^	20 ms, 10 ms [26]
Refractory period for E, I neurons	*t*_refratory_^*E*^, *t*_refratory_^*I*^	*2* ms, 1 ms [26]
Reversal potential of E, I neurons	*E* ^*E*^, *E*^*I*^	0 mV, -70 mV [26]
Input conductance from background to E, I neurons	*g* ^*EO*^, *g*^*IO*^	0.05, 0.05 (normalized by leakage conductance)
Input conductance from E to E, I to E, E to I, I to I	*g* ^*EE*^, *g*^*EI*^, *g*^*IE*^, *g*^*II*^	0.1, 0.6, 0.84, 0.48 (normalized by leakage conductance)
Decay time constant of excitatory, inhibitory current	*τ*_*d*_^*E*^, *τ*_*d*_^*I*^	3 ms, 8 ms
Rising time constant of excitatory, inhibitory current	*τ*_*r*_^*E*^, *τ*_*r*_^*I*^	0.5 ms, 0.5 ms [26]
Facilitation time constant in STP	*τ*_*F*_	1500 ms [41]
Depression time constant in STP	*τ*_*D*_	200 ms [41]
Initial neural transmitter release probability in STP	*U*	0.2 [41]
Learning rate for LTP	*A*	0.001 [21]
Learning rate for LTD	*B*	0.001 [21]
Transmitter plasticity strength	*δ*_1_	1 × 10^−5^ [19]
Learning rate for heterosynaptic plasticity	*β*	0.01 [19]
Heterosynaptic plasticity parameter	$\tilde{w}$	0.1
Characteristic time constant for synaptic trace	*τ*_STDP_	20 ms [21]
Characteristic time constant for slow synaptic trace	*τ*_STDP_slow_	100 ms [21]

## Data Availability

The data used to support the findings of this study are available from the corresponding author upon request.

## Supplementary Materials

Supplementary Materials The following supplementary materials are available for this paper. Figure S1: instantaneous firing rate distribution of neurons in different states. Figure S2: result of final stable synaptic strengths on manipulations combining spike time randomization and empty bin inserting to modify spike synchrony and rate. Figure S3: results of artificial generated network structure.
