# Supplementary material for: Gamma Oscillations Facilitate Effective Learning in Excitatory-Inhibitory Balanced Neural Circuits
Source: Neural Plast. 2021 Jan 20;2021:6668175. doi: 10.1155/2021/6668175 (PMC7840255; doi:10.1155/2021/6668175)
Supplement: Supplementary Materials — The following supplementary materials are available for this paper. Figure S1: instantaneous firing rate distribution of neurons in different states. Figure S2: result of final stable synaptic strengths on manipulations combining spike time randomization and empty bin inserting to modify spike synchrony and rate. Figure S3: results of artificial generated network structure. [file 6668175.f1.docx]

Supplementary Material:

Gamma oscillations facilitate effective learning

in excitatory-inhibitory balanced neural circuits

Kwan Tung Li, Junhao Liang and Changsong Zhou*

[*Correspondence: cszhou@hkbu.edu.hk](mailto:*Correspondence:%20cszhou@hkbu.edu.hk)


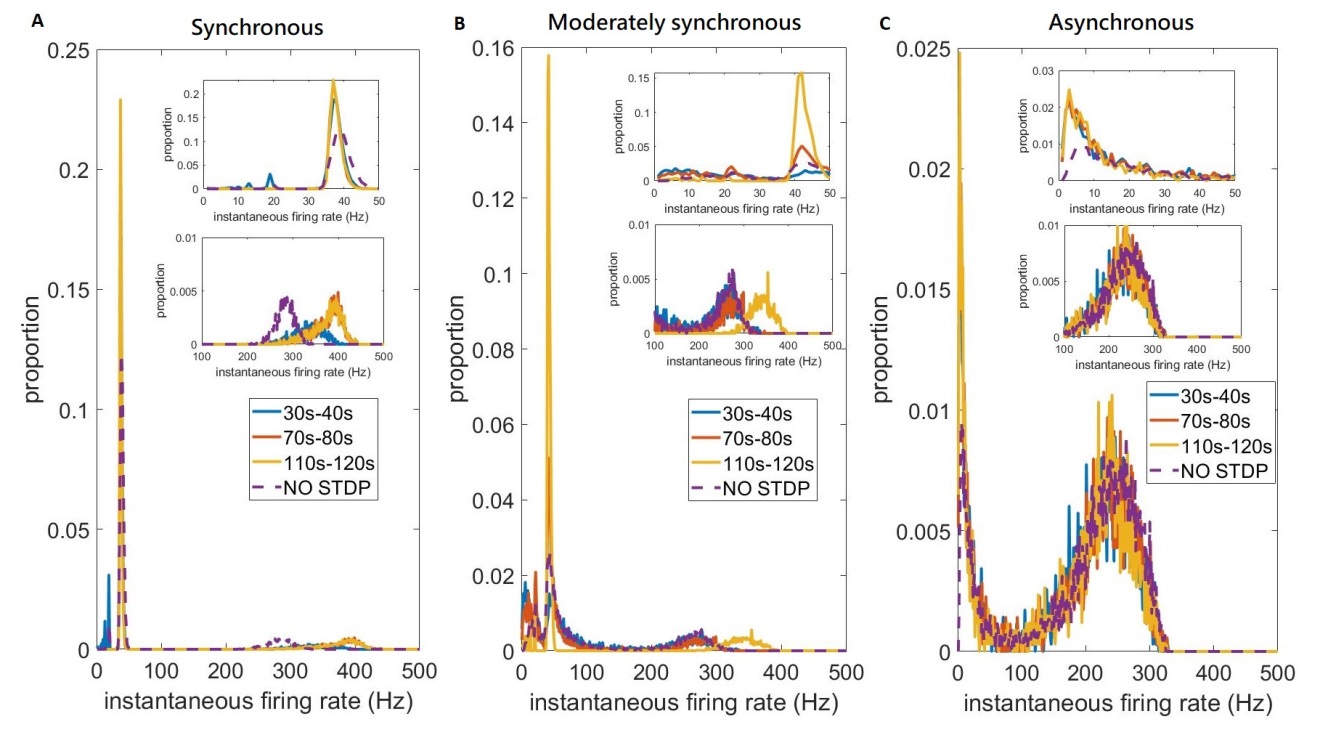


Figure S1. Instantaneous firing rate distribution of neurons in different states. Instantaneous firing rate of neurons is defined by the inverse of inter spike interval. Inserts show the amplified plot at 0-50Hz and 100- 500Hz respectively for clearer comparison. The results of circuit with plasticity in different learning stages from 30s~40s, 70s~80s, 110s~120s, the results of circuit without plasticity are plotted in different curve types. (A): Synchronous state. $\tau_{d}^{E}=6ms.$ (B): Moderately synchronous state. $\tau_{d}^{E}=10ms$. (C): Asynchronous state. $\tau_{d}^{E}=90ms.$ The other parameters are set as $\tau_{d}^{I}=8ms, As=3.5.$


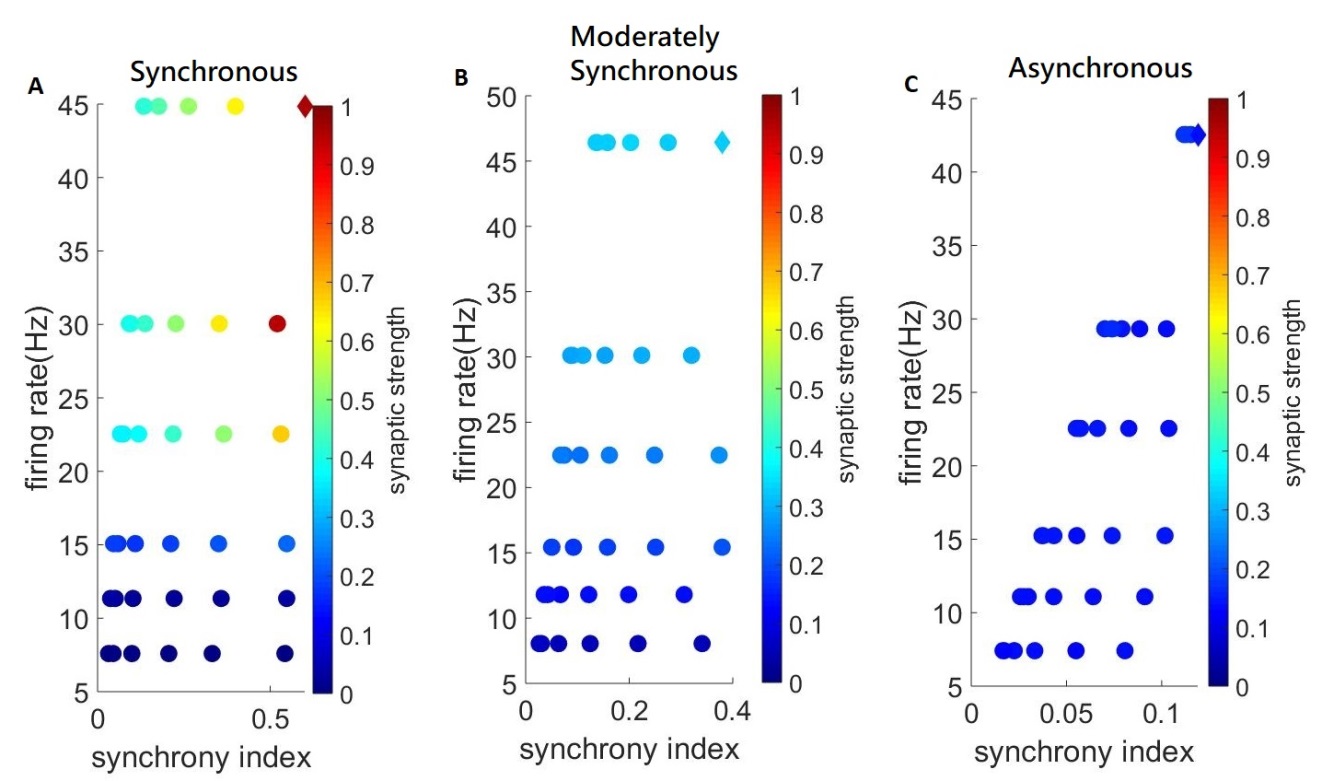


Figure S2. Result of final stable synaptic strengths on manipulations combining spike time randomization and empty bin inserting to modify spike synchrony and rate. The result without manipulation is indicated by diamonds. From right to left, the amount of randomization is 0%, 20%, 40%, 60%, 80% and 100%. From top to bottom, the empty bin inserted are 0ms, 5ms, 10ms, 20ms, 30ms, 50ms. (A): Synchronous state with $\tau_{d}^{E}=6ms$. (B): Moderately synchronous state with $\tau_{d}^{E}=10ms$. (C) Asynchronous state with$\tau_{d}^{E}=90ms$. Other parameters are $\tau_{d}^{I}=8ms,As=3.5$.


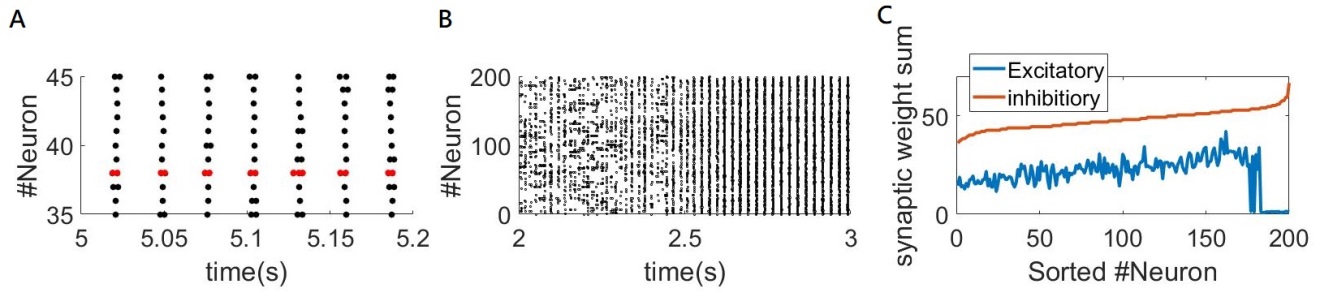


Figure S3. Results of artificial generated network structre. (A): Raster plot (only 9 neurons around #38 are shown) picking out one neuron (#38) in learnt synchronous circuit. We increased the excitatory input to one of the picked neuron (#38). After this increase of input strength, this neuron tends to spike in bursts. Parameters are set as $\tau_{d}^{E}=6ms, \tau_{d}^{I}=8ms, As=3.5 .$ Red dot are spikes of the picked out neuron. (B-C): Result of randomly generated circuit without STDP. (B): Raster plot result. Synchronous starts at around 2.5s. (C) Incoming synaptic weight sum of the generated circuit. X-axis is sorted in ascending order of excitatory synaptic weight sum.
